# Supplementary material for: The Combination of Loss of ALDH1L1 Function and Phenformin Treatment Decreases Tumor Growth in KRAS-Driven Lung Cancer
Source: Cancers (Basel). 2020 May 28;12(6):1382. doi: 10.3390/cancers12061382 (PMC7352727; doi:10.3390/cancers12061382)
Supplement: Supplementary file 1 [file cancers-12-01382-s001.pdf]

Supplementary Materials

The combination of loss of ALDH1L1 function and phenformin treatment decreases tumor growth in *KRAS*-driven lung cancer

Seon-Hyeong Lee, Yoon Jeon, Joon Hee Kang, Hyonchol Jang, Ho Lee and Soo-Youl Kim

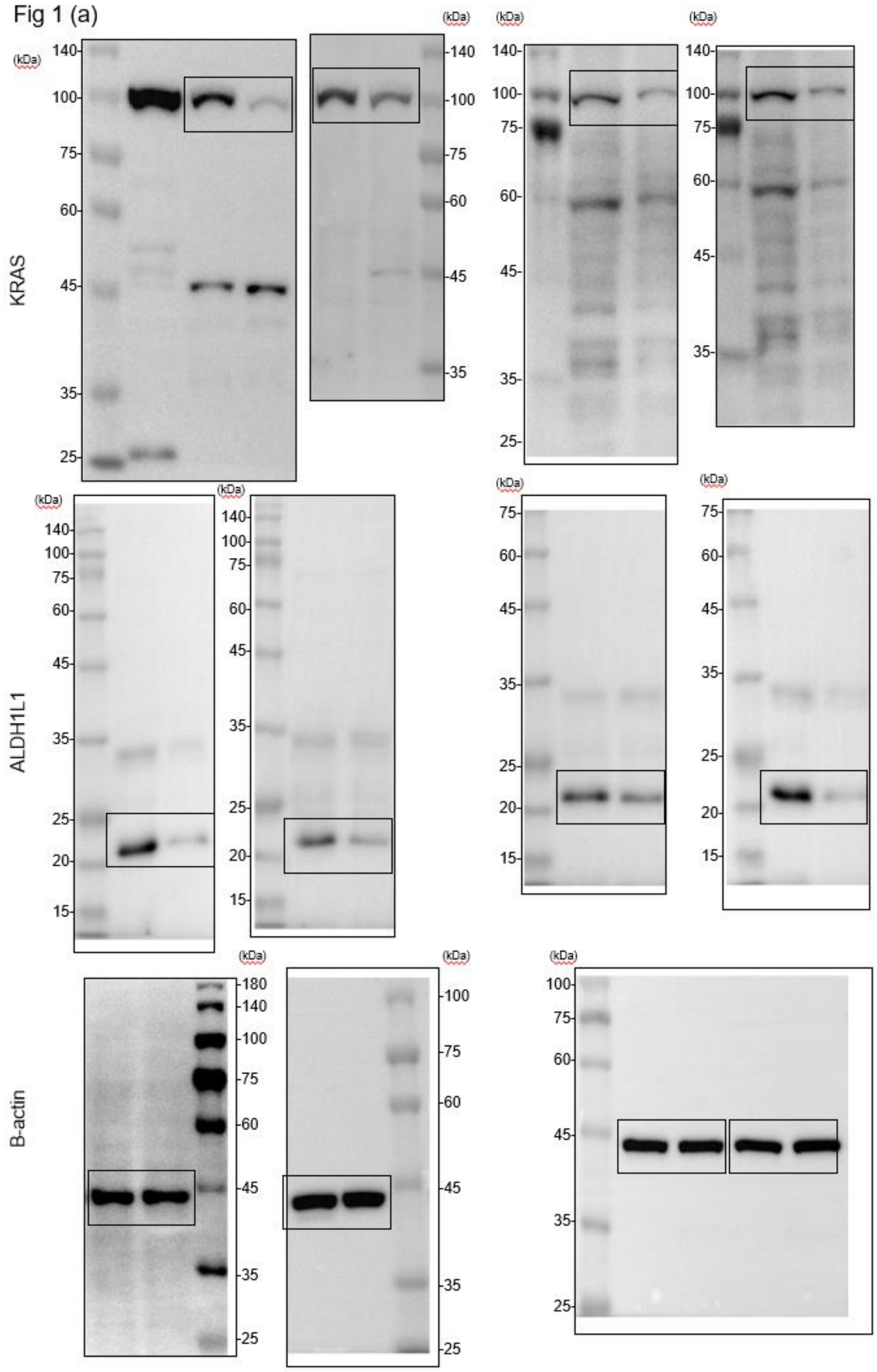

Figure S1. Detailed information about western blot in Figure 1.

Fig 3 (a)

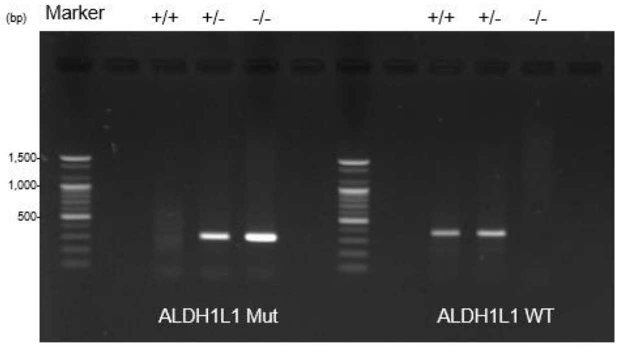

Fig 3 (b)

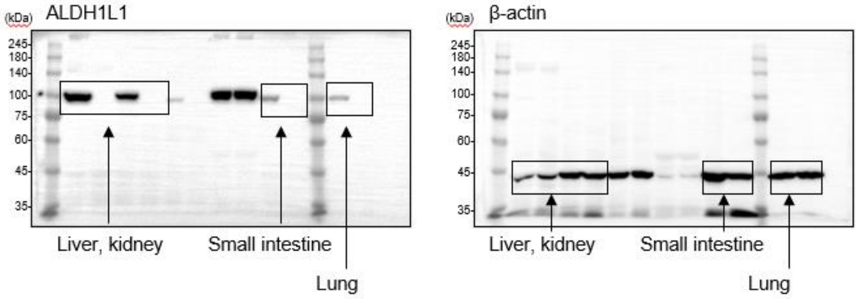

Figure S2. Detailed information about western blot in Figure3.

© 2020 by the authors. Licensee MDPI, Basel, Switzerland. This article is an open access article distributed under the terms and conditions of the Creative Commons Attribution (CC BY) license (http://creativecommons.org/licenses/by/4.0/).
